# Patient complexity and genotype-phenotype correlations in biliary atresia: a cross-sectional analysis

**DOI:** 10.1186/s12920-017-0259-0

**Published:** 2017-04-17

**Authors:** Guo Cheng, Patrick Ho-Yu Chung, Edwin Kin-Wai Chan, Man-Ting So, Pak-Chung Sham, Stacey S. Cherny, Paul Kwong-Hang Tam, Maria-Mercè Garcia-Barceló

**Affiliations:** 1 Department of Surgery, 1/F Hong Kong Jockey Club Building for Interdisciplinary Research, 5 Sassoon Road, Pokfulam, Hong Kong; 2 Department of Psychiatry, The University of Hong Kong, Hong Kong, SAR China; 3 Center for Genomic Sciences, Hong Kong, SAR China; 4 Centre for Reproduction, Development, Growth of the Li Ka Shing Faculty of Medicine, Hong Kong, SAR China; 5 State Key Laboratory of Brain and Cognitive Sciences, The University of Hong Kong, Hong Kong, SAR China; 6 Department of Surgery, the Chinese University of Hong Kong, Hong Kong, SAR China

**Keywords:** Copy number variant, Rare complex disease, Genotype-phenotype correlation, Network

## Abstract

**Background:**

Biliary Atresia (BA) is rare and genetically complex, and the pathogenesis is elusive. The disease course is variable and can represent heterogeneity, which hinders effective disease management. Deciphering the BA phenotypic variance is a priority in clinics and can be achieved by the integrative analysis of genotype and phenotype. We aim to explore the BA phenotypic features and to delineate the source of its variance.

**Methods:**

The study is a cross-sectional observational study collating with case/control association analysis. One-hundred-and-eighty-one type III non-syndromic BA patients and 431 controls were included for case–control association tests, including 89 patients (47.19% males, born June 15th, 1981 to September 17th, 2007) have detailed clinical records with follow-up of the disease course (median ~17.2 years). BA-association genes from the genome-wide gene-based association test on common genetic variants (CV) and rare copy-number-variants (CNVs) from the genome-wide survey, the later comprise only CNVs > 100 kb and found in the BA patients but not in the local population (*N* = 1,381) or the database (*N* = 11,943). Hereby comorbidity is defined as a chronic disease that affects the BA patients but has no known relationship with BA or with the BA treatment. We examined genotype-phenotype correlations of CNVs, connectivity of these novel variants with BA-associated CVs, and their role in the BA candidate gene network.

**Results:**

Of the 89 patients, 41.57% have comorbidities, including autoimmune-allergic disorders (22.47%). They carried 29 BA-private CNVs, including 3 CNVs underpinning the carriers’ immunity comorbidity and one *JAG1* micro-deletion. The BA-CNV-intersected genes (*N* = 102) and the CV-tagged genes (*N* = 103) were both enriched with immune-inflammatory pathway genes (FDR *q* < 0.20), and the two gene sets were interconnected (permutation *p* = 0.039). The molecular network representing CVs and rare-CNV association genes fit into a core/periphery structure, the immune genes and their related modules are found at the coherence core of all connections, suggesting its dominant role in the BA pathogenesis pathway.

**Conclusions:**

The study highlights a patient-complexity phenomenon as a novel BA phenotypic feature, which is underpinned by rare-CNVs that biologically converge with CVs into the immune-inflammatory pathway and drives the BA occurrence and the likely BA association with immune diseases in clinics.

**Electronic supplementary material:**

The online version of this article (doi:10.1186/s12920-017-0259-0) contains supplementary material, which is available to authorized users.

## Background

Extra-hepatic biliary atresia (or biliary atresia, BA [OMIM 210500]) is a rare disease that manifests within the first few months of life and is characterized by an idiopathic, destructive, inflammatory process affecting both intra- and extra-hepatic bile ducts. BA is diagnosed anatomically through operative cholangiograms, in which visualized obstruction of the extra-hepatic bile ducts confirms the disease clinical diagnosis [1]. However, clinical manifestations of the disease, e.g. symptom onset, treatment response, and the disease outcome [2], vary among patients, whether BA is an end phenotype of multiple disease process has been questioned [2]. Elucidating the extent and the source of the disease phenotype variance thus to develop an effective disease treatment strategy is one major task in both basic and clinical studies of BA.

BA can be categorized according to the anatomical features, i.e. the coexistence with systemic anomalies (syndromic BA versus non-syndromic BA), or the anatomical location of the atretic segment (type I and II versus type III BA). Yet most patients are non-syndromic and type III BA, which comprises ~95% of BA cases in Asians. The type III BA can be corrected by Kasai operation, but even with successful bile drainage in a majority of the cases, only about 10% of the patients can have a long-term survival with their native livers, which reflects that the BA phenotype can be more complex beyond the clinical diagnosis based on the anatomy.

Etiopathogenic stratification of BA is yet to be practical. The disease etiology involves multiple plausible risk factors, including viral infections, developmental anomalies, and immunity disorders, yet none of these has consolidated evidence with mechanistic proof for their causal relationship with BA. In-depth exploration on the disease endo-phenotype is hurdled by our lack of knowledge of the liver development, especially that at the perinatal period when BA occurs. Meanwhile, results from the clinical epidemiological studies can be confusing due to the rarity and the complex pathogenic mechanism of BA.

Genetic variants are associated with BA [1]. We previously showed that common variants with footprints of genetic-environmental interaction confer risk of BA. Also, a handful of rare mutations has been detected in syndromic BA or non-syndromic BA with severe clinical manifestations [3–7]. Overall, BA represents a rare disease with a complex genetic architecture. We anticipate that the phenotypic impact of common and rare genetic variants, especially the rare variants with major effect size, can be explored to decipher the BA phenotypic variance and/or heterogeneity.

Trying to bridge the genotype-phenotype gap, the challenge of the genetic analysis on BA are the uncertainty of the BA phenotype and its complexity, the low survival rate that makes pedigree data scarce, and also the power of discovery issue given the rarity of the disease. Although exploration on the developmental candidate gene mutation in BA was fruitful, its value is limited as individual finding that may represent only a heterogeneous group of patients [5–7].

For a genome-wide exploration of the impact of rare genetic variants on BA, the study of large genomic variations, i.e. copy number variants (CNVs), is ideal to start with, given its technical advantages, the straightforwardness regarding functional interpretation, and importantly, the fact that BA does co-occur with chromosomal diseases, which implicate a possible role of gene dosage variations in BA aetiology [8].

This study started with a genome-wide survey on the association of rare CNVs. The BA clinical manifestations reviewed with a median clinical follow-up of ~17 years post-Kasai operation, as we think that in-depth phenotyping of the disease can be achieved by reviewing the patients’ chronic disease course. After the dissection of the CNV-genotypes--phenotype correlations (GPCs), the rare CNVs and the GPCs were collated with common genetic variants to examine their role in the disease genetic architecture. The output would not only enhance our understanding of the BA clinical phenotype variance/heterogeneity but also can provide a framework of dissecting the GPCs of a rare complex disease.

## Methods

### Subjects

Up to March 2014, the clinical records of patients had been stored in the computer system administered by the government hospital authority and were retrospectively reviewed in this study. Patients in the genome-wide association study on BA were recruited from August 1st, 2005 to October 31st, 2007 [9]. After the pre-CNV-calling sample quality control (QC), 89 Chinese patients from Hong Kong remained and were reviewed for their full clinical records, who represent non-syndromic type III Southern Chinese BA patients with long-term survivals with/without their native livers. The patients (42 males and 47 females) were born from June 15th, 1981 to September 17th, 2007). Up to March 2014, clinical follow-up of these patients had been conducted for a median of 17 years 2 months (from 6.29 to 32.57 years, interquartile range, IQR ~ 10.00 to 22.61 years). Thirty patients had liver transplantation after Kasai operation. After the recruitment two patients passed away between 2006 and 2013, one patient was from liver failure while waiting for the transplantation and the other patient from intractable seizures at 2 years after liver transplantation.

Two sample sets with genome-wide CNV profiles were employed as the reference of novelty evaluation of the CNVs discovered in BA: 1). Database of Genomic Variant (DGV) data (http://dgv.tcag.ca/dgv/app/home; last accessed in July 2014) that includes 11,943 individuals from different ethnic groups genotyped on various chips with different coverage; 2) 1,381 Chinese Han normal individuals genotyped on Illumina Human 610-Quad BeadChips [10].

Patients recruited in the previous GWAS study but do not have accessible full clinical follow-up records are not included in the analysis. The control samples in the GWAS were not included in this study because of the difference on copy number genotyping platform [9].

The gene-based genome-wide association analysis employed the 289,118 single nucleotide polymorphisms (SNPs) genotyped on 181 type III isolated BA patients and 481 matched controls, which had undergone SNP-based QCs as reported [9]. Principal component analysis was performed as described and was incorporated in the association test [9].

### CNV calling and quality control

Intensity measurements from all autosomal SNP probes and non-polymorphic copy number (CN) probes were used to identify deletions and duplications based on 3 software, Birdseye [11] (of Birdsuite v1.5.5 package), PennCNV [12] and iPattern [13]. Only concordant calls by all the three programs with the segments ≧100 kb and covered by at least 5 probes were considered for the downstream analysis. As the MHC region (chr6: 25, 000, 000–35, 000, 000) and the centromeric and telomeric regions are likely to harbor spurious CNV calls we removed any CNV segment with >50% overlap with these regions. Details of the calling process are described in Additional file 1: Supplementary methods and Figure S1.

We termed a CNV as a BA-CNV in the manuscript if it was not found in the reference dataset (11,943 DGV samples, and the 1,381 Chinese samples), which was defined technically as a CNV that did not overlap with >50% of the CNV length with any reference CNVs at the same direction as for dosage effect. The BA-CNVs then were validated by quantitative real-time Polymerase Chain Reaction (PCR) (ABI Prism 7900 Sequence Detection System; Applied Biosystems) using TaqMan® Copy Number Assay (Additional file 1: Supplementary materials).

For function interpretation, boundaries of the called CNV regions were extended by 30 kb both downstream and upstream, and the CNVs were considered to affect the dosage of a gene if they intersected with RefSeq exonic regions. Genotype/gene and phenotype associations were explored in the DisGeNet portal (http://www.disgenet.org/) and DECIPHER (https://decipher.sanger.ac.uk/). For gene-phenotype associations, only those with genomic association proof were considered, and all disease associations of interesting gene/genotypes were then verified by a literature search in NCBI (http://www.ncbi.nlm.nih.gov/).

### Gene-based genome-wide association study

Gene-based association analysis congregates the effect of multiple SNPs within each genetic locus to elucidate the disease-associated genes, for this purpose KGG (Knowledge-based Genome-wide Genetic association) software as a gene-based association test program was applied to perform the test [14]. In addition, liver and lymphoblastoid expression quantitative trait loci (eQTL) in the GTEX database were also employed (http://www.gtexportal.org/; last assessed in December 2014). We arbitrarily selected genes with eQTL SNP BA-association *p* < 5×10^−3^ as BA association genes (Additional file 2: Tables S2–3 and Additional file 1: Figure S2).

### Interconnectivity of BA-CNVs and SNPs, and the genetic network

Four hundred and forty canonical pathways from KEGG, BIOCARTA, PID and ST were retrieved from GSEA (http://software.broadinstitute.org/gsea; last assessed in November 2015), and only those with 20 to 200 component genes were included. The intersection of the genes affected by the novel CNVs with the pathway gene sets was tested using 1 side Fisher’s exact test for statistical significance. For SNPs, enrichment in the functional gene sets was tested by i-GSEA program [15]. Association significance under the multiple-testing framework was evaluated through Benjamini and Horchberg false discovery rate (FDR) *q* value [16], q < 0.05 is statistically significant, while *q* < 0.20 would be considered noteworthy.

The interconnectivity test between the two gene sets tagged by CNVs and SNPs as well the following gene network construction was based on the protein-protein interaction (PPI) meta database InWeb (http://www.cbs.dtu.dk/suppl/dgf/heart_developmental_networks/) [17, 18], which combines the interaction knowledge from multiple PPI databases and only confident interactions were kept for analysis. Among the 350,006 PPI in the database, we selected 129,341 PPIs with confidence score ≥0.01 (3,623 self-loops, i.e. proteins that connect with themselves, were removed). Connectivity among encoded proteins is defined as the number of interactions that connect the candidates, while non-coding genes were excluded. Statistical significance of the connectivity was evaluated by a random gene sampling of 10,000 times, the connection/interaction with an empirical *p* < 0.05 means that less than 5% of the sampled gene sets of the same size establish more connectivity than the candidate genes do, thus suggest significant interconnectivity of the candidate genes. Finally, in building the network we allowed at the most 3 interactors in the graph to link two candidate genes (~proteins) even if they do not have direct interactions, a random cut off would be applied if more than 3 interactors exist between the pairs of candidates.

## Results

### Phenotype: the Non-syndromic BA is not isolated

The gene/genotype and phenotype correlation analysis include 89 patients with a full clinical record and 86 out of the 89 patients with confident CN callings (Additional file 1: Supplementary methods). Retrieval of patients’ long-term medical record (median ~ 17.2 years) showed that up to 41.57% of the non-syndromic type III BA patients have been affected by other chronic conditions (Table 1). Hematological disorders affected 11.24% of the patients, in which Glucose-6-Phosphate Dehydrogenase (G6PD) deficiency occurred 14.29% of the BA male patients, whereas ~4.8% of males in the Hong Kong have the condition [19]. Chronic autoimmune-atopic diseases followed to affect 22.4% of the patients after the Kasai procedure, including autoimmunity occurred in 5.6% of the patients before liver transplantation, comparing to the disease prevalence of 3.1% in the local population, while long-lasting or recurrent atopic conditions remarkably have attacked more than one-sixth of the patients.

**Table 1:** Frequency of extra-hepatic conditions in BA patients

Category	Extra-hepatic diseases	N. patients	Prevalence (%)	Overall prevalence (%)
Allergic diseases	Asthma	7	7.87	16.85
	Eczema	9	10.11	
Autoimmune diseases	Psoriasis	2	2.25	5.62
	Juvenile arthritis	1	1.12	
	Hashimoto thyroiditis	1	1.12	
	Type 1 diabetes mellitus	1	1.12	
Hematological diseases	G6PD	6	6.74	11.24
	Alpha thalassemia trait	2	2.25	
	Thrombocytopenia	1	1.12	
	Pancytopenia	1	1.12	
Neural& Psychiatric diseases	ADHD^a^	2	2.25	7.87
	Epilepsy	3	3.37	
	Depression	1	1.12	
	Mental retardation	1	1.12	
Structural malformations	Intestinal malrotation	1	1.12	5.62
	Pulmonary atresia with VSD^a^	1	1.12	
	Temporal AVM^a^	1	1.12	
	Vesicoureteric reflux grade IV	1	1.12	
	Jejunal atresia	1	1.12	
Metabolic diseases	Hyperlipidemia	1	1.12	
	Congenital hypothyroidism	1	1.12	
Others	Sleep apnea	3	3.37	4.49
	Deviated nasal septum and hypertrophy of nasal turbinate	1	1.12	

^a^ *G6PD* glucose-6-phosphate dehydrogenase, *ADHD* attention deficit hyperactivity disorder, *VSD* ventricular septal defect, *AVM* arteriovenous malformation

Except for the structural developmental anomaly has been described in BA and affects 5.62% of our patients (non-syndromic BA because they carry only one such anomaly), the relationship of the comorbidities with BA can be a coincidence from a random chance or can suggest disease-disease associations driven by the sharing of disease etiopathogenesis. The remarkably high incidence of comorbidities in the young BA patients, indeed, support a possible association between BA and the comorbidities while bringing up the patient complexity issue of the non-syndromic BA patients that requires a follow-up study.

### Genotype: BA-CNVs and genotype-phenotype correlations

Twenty-six of the 86 patients carry at least one CNV that each spans more than 100 kb and has been found in patients only referring to the CNV profile in the general population (Table 2). The CNV burdens between the patients with comorbidities and those without are not significantly different (Chi-Square *p* = 0.71). Yet interestingly, concurring with the high incidence of immunity disorders in the patients, the BA-CNV regions are enriched with immunity-related genes (Table 4; FDR *q* < 0.10). Also, the fact that the novel variants carried by different BA patients tend to be immunity related supports the association of the BA-CNVs with BA. Moreover, as we analyzed the CNV profile of each patient and their clinical record, we found 3 BA-CNVs encompassing genes that are associated with the extra-hepatic conditions that the 3 patients have (Table 3).

**Table 2:** Stats of BA-CNVs

	Deletions	Duplications
No. CNVs	6	21
No. Affected Genes	26	76
Average Length	187 kb	459 kb
Median Length	292 kb	458 kb

**Table 3:** CNV genotypes that correlate with BA patients’ clinical phenotype

Patients	Comorbidity	CNV location	Gene	Human phenotype
BA99	Diabetes Mellitus type 1	16:81947921-82095870 (dup)	*PLCG2*	PLCG2-associated antibody deficiency and immune dysregulation; **diabetes mellitus type 1** [33]
BA44	Hashimoto thyroiditis	18:7080664-7584939 (dup)	*PTPRM*	**Autoimmune disease** [34, 35]
BA68	Eczema	18:55546006-56018494 (dup)	*NEDD4L*	**asthma**; metabolism; hypertension disease [36, 37]
BA123	nil	20:10533666-10694525 (del)	*JAG1*	Alagille syndrome, **BA** [7]

In addition, we discovered one *de novo* microdeletion intersecting *JAG1* in a BA patient (BA123; Additional file 2: Table S1), who was clinically diagnosed as type III BA by intra-operative cholangiogram study. The patient underwent liver transplantation due to liver failure before 1 year old but has no ductopenia was noted at the time of diagnosis. We consider the *JAG1* deletion as a known genotype to BA as it has been described in the DECIPHER database (https://decipher.sanger.ac.uk/), although *JAG1* is best known for its role in NOTCH signaling and liver development and its association with Alagille syndrome [20].

All the CNVs were validated by TaqMan probes. Except for the *JAG1* deletion, all were inherited from unaffected parents.

Despite the small scale of the CNV and GPC discovery, our finding suggests that indeed these rare BA-CNVs can connect with the clinical manifestation of the disease. However, it is intriguing to speculate whether the patient who carries the *JAG1* deletion and those who carry immune gene duplication/deletions represent two distinct phenotypes of BA, as it seems that the function of those two genotypes diverges from each other. To address the issue, we collated the rare CNVs with the BA candidate genes generated from common variants test in order to understand the role of BA-CNVs in the context of the BA genetic architecture.

### The BA candidate genes from genome-wide common variants association test

Through the gene-based genome-wide association test using SNPs, we obtained 103 candidate genes with genome-wide significance (Additional file 1: Figure S2). The comparison between the 103 genes tagged by SNP associations with the genes encompassed by BA-CNVs (*N* = 102) revealed one overlapped gene *S100A13* (Additional file 2: Table S1 and S2), which has decreased expression in the BA population (lymphoblastoid), and is deleted in one BA patient. The encoded protein S100A13 belong to the S100 protein family, which participate in the regulation of protein phosphorylation, Ca^2+^homeostasis, the dynamics of cytoskeleton constituents, enzyme activities, and the inflammatory response [21]. S100A13 itself is required for stress-induced IL-1A and FGF1 exportation and it is involved in inflammation and angiogenesis [22].

SNP-based pathway enrichment was tested by synthesizing the SNP associations into each functional gene set, which only yielded the IL1R-related inflammatory pathway with *p* = 1.3×10^−3^, FDR *q* = 0.16 of suggestive disease association (Table 4). Yet, the fact the BA-CNV-affected genes and the SNP-tagged genes that both are most enriched with immunity-related genes indicates possible connections between the two gene sets.

**Table 4:** Gene set enrichment analysis on genes encompassed by CNVs and SNPs

Gene set^a^	*P* ^*b*^	*FDR q* ^*b*^
Genes encompassed by BA-CNVs		
Antigen_processing_ubiquitinaination_proteasome_degradation	1.05 × 10^−3^	0.070
Signaling_by_the_B_cell_recepceptor_BCR	2.39 × 10^−3^	0.096
Class_I_MHC_mediated_antigen_processing_presentation	1.95 × 10^−3^	0.096
Genes tagged by SNP associations		
IL1R_pathway	1.30 × 10^−3^	0.16

^**a**^IL1R pathway in the listed gene sets are from BIOCARTA database, the others enlisted are from REACTOME; ^b^The association stats on CNVs and SNPs were obtained from different statistical test as described in methods

### Biological convergence of the rare and the common genetic variants

**Fig. 1:**
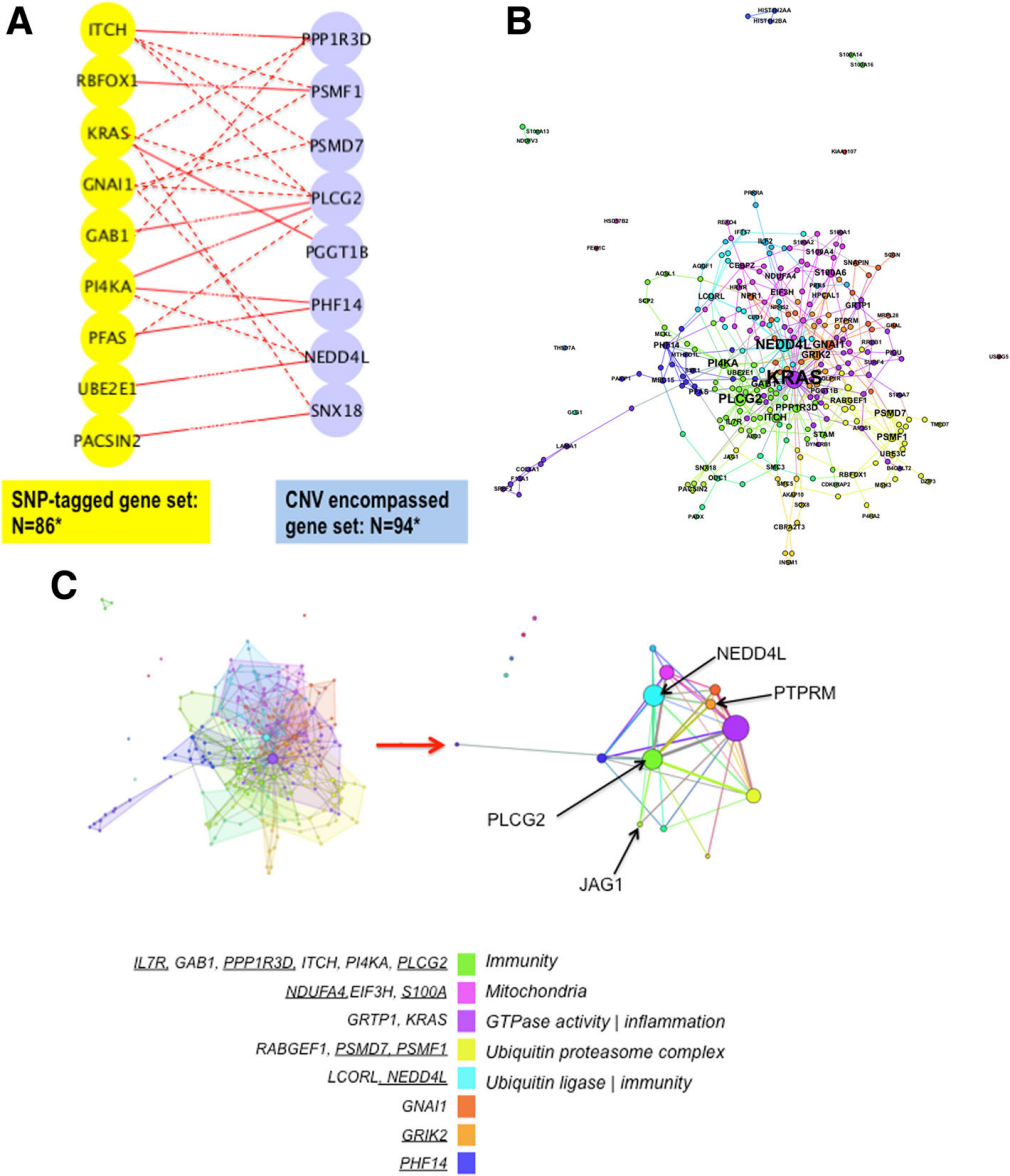
The molecular network of BA. **a** The direct interactions between the genes tagged by common and rare genetic variants are shown by solid lines while indirect interactions by dashed lines. Interactions that are only mediated by interactors are not shown. *Number of protein-coding genes that have interactions in the interactome database that we employed. **b** The protein network. Plotted by Gephi via the Force Atlas the ‘power-based’ approach (https://gephi.org/). Each node designates a protein encoded by the BA candidate genes (labeled), or an interactor that mediates the connection of two candidates, the edge of the network represent the PPIs. Colors represent the module classes of the nodes that are set to not overlap with each other while the node size is set to be proportional to its centrality in the network. **c** The core-periphery structure of the network. *Left upper panel*: at the center of the network the functional modules closely connect and seem to pile upon each other, while at the periphery the connection is sparse; *right upper panel*: the network of meta-nodes that grouped all the nodes in each module. The size of the meta-node is proportional to its centrality. The major meta-nodes belong at the core of the network and are connected with triangles between each other contrasting with the sparsely located periphery nodes; *lower panel*: the color column denotes the functional modules, on its *left* side are the candidate genes of top centralities with BA-CNV encompassed genes underlined, on the *right* side are the function annotation of each module that was inferred from the shared functionality of its representative genes

We then tested the interconnectivity between the BA-CNV encompassed genes (*N* = 102) and the SNP-tagged genes (*N* = 103) based on protein-protein interactions (PPIs). Excessive interactions between the encoded proteins of the two gene sets are observed (Permutation = 10,000, empirical *p* = 0.041; Fig. 1a), so are the overall interactions among all the genes as they were pooled (empirical *p* = 0.037).

In the network built from the CNV and the SNP tagged genes, we first looked for the nodes that interact with each other more frequently that with others, i.e. modules, we then interrogated the functionality of each module based on the ranked component genes. We found that the immunity-related genes cluster at the largest module (Gene = 13, including N_CNV_ = 5, N_SNP_ = 8) [23], and is located at the center of the network together with 3 other closely connected modules showing cohesiveness in function annotation structure (Fig. 1b-c; Additional file 1: supplementary materials with numerical parameters of the network).

Overall the network exhibits a cohesive core and sparsely connected periphery structure (Fig. 1b-c). The 3 genes encompassed by BA-CNVs that show immunity related GPCs are located at the core of the network (Table 3, Fig. 1c) [24]. Meanwhile, despite its interaction with the core as mediated by the interactor NOTCH1 (Fig. 1b), JAG1 is found at the periphery of the network. The same core-periphery location is found of the modules of these genes (Fig. 1c). Considering the network as a complex dynamic system that evolves with the addition/reduction of candidate gene/genotypes or exogenous environmental hazards, the core can be regarded as a stable facilitator in the system of pathogenesis catering the variance among patients, whereas JAG1 could just represent an individual case finding.

S100A13 interacts with NDUFV3 (tagged by SNPs), a NADH dehydrogenase that belongs to the accessory subunit of the mitochondrial membrane respiratory chain (Fig. 1b). Other *S100* members in the same CNV segment with *S100A13* are more closely connected with each other and are connected with NDUFA4, a NADH dehydrogenase in the respiratory chain (Fig. 1c). The connection of S100 with the NADH dehydrogenase and its role in Ca^2+^ homeostasis indicate that its involvement in BA can be related to mitochondrial function. We speculate whether this could be linked with the over-representation of G6PD deficiency in BA. Yet indeed, the interconnections among the S100 members driven by a single BA-CNV invite our concerns that the importance of the module in the BA genetic network can be inflated and warrant further validations.

## Discussion

The comorbidities of BA used to be recognized as a list of developmental anomalies that present before or at the BA diagnosis, which suggests that developmental defects can be associated with BA [25–27]. Due to the disease severity and rarity, the BA comorbidities that appear at the late phase of the disease course can be neglected. While humans are more likely to be affected by chronic diseases as we age, that 41.52% of the BA patients of a median age ~17 years are affected by additional chronic diseases is extraordinary, which reflects the patient complexity issue of BA that not only warrants follow-ups of patients’ life quality in the clinical settings but also can represent a novel phenotypic feature of the non-syndromic BA.

Indeed, comorbidities can indicate a disease-disease association, whereby statistical test on the relative risk of the disease incidence as well as disease etiopathogenic interrogation represents the two complementing approaches to dissect such connections. Intriguingly, the disease-disease association can open a new window to interpret the diseases phenotype like BA, as whereas the disease pathology itself is difficult to delineate due to our lack of knowledge of the human liver biology, the disease identity can be elucidated from its connections with other diseases.

In our comorbidity list based on the 89 patients with ‘full’ clinical record and long-term follow-ups, about 5.6% of BA had autoimmunity disorders before LT compared to the 3.1% disease incidence in the local population, which coincides with a previously report in the Caucasian population where 44% of BA patients have first-degree relatives affected with autoimmune disorders [26]. Altogether 22.47% of our BA patients have been affected with chronic immune-atopic diseases, these diseases frequently require steroids treatment that overlaps with the post-portoenterostomy steroid administration to BA [28]. Although relative risks detected for these comorbidities are very modest to suggest the association of BA with these immunity disorders [29], the findings reminded us that immunity deregulation has been postulated to play a major role in BA pathogenesis [30], which notably, has been supported by transcriptome analysis and candidate gene-based association tests [31].

Meanwhile, the BA-CNVs in these patients also are associated with immunity-related phenotypes. With the shortlisted 29 BA-CNVs that each spans >100 kb and are found in BA only, we tried out the ‘genotypes first’ approach [32], whereby we interpreted the functional consequences of BA-CNVs according to the phenotype they are associated with. Interestingly, we found that BA-CNVs can correlate with the comorbidities in the patients. Moreover, the immune-inflammatory processes consistently proved to dominate the molecular network underpinning the BA pathogenesis.

Following the GPC discovery, we performed the genome-wide gene-based SNP association test to elucidate the BA candidate genes that may provide us a framework of the BA genetic landscape, and with its reference, the role of the GPCs and the BA-CNVs can be interrogated. Common variants require much fewer samples for the power of association discovery, and we got 103 genes though the cases control association test. While the connection between the CNV and the SNP at the molecular level indirectly fortifies the association of BA-CNVs with BA (permutation test *p* = 0.041), it also granted us a chance to explore further about our GPC findings.

The positive GPCs are small scaled but are interesting that *JAG1* known to the biliary embryonic development and the other 3 genes known to immunity disorders are enlisted, which indicate the possible existence of disease heterogeneity. We then located these interesting genes in the network of BA as a genetically complex disease, which proved again the core position of immunity-inflammation genes in BA coordinating the pathogenic process, which otherwise can be a difficult challenge due to our lack of knowledge about liver immunobiology, especially that in the developmental stages.

Meanwhile ‘negative’ GPCs are expected. We believe that the outlined GPCs between the BA-CNVs and the immunity comorbidities of BA may just showcase the link between the genotype and the disease phenotype. Actually, only about 40% of the human genes have phenotype coverage according to the OMIM and ClinVar, the two most popular genetic association databases. Even for genes with known disease associations, the variance of expression and the allelic heterogeneity is the norm. The genetic network hereby ‘smooths’ the bias and incompleteness of our knowledge, as the functionality of a gene can be inferred from its interacting genes and location in the network.

Overall, we admit there are limitations of the study as a cross-sectional analysis. The comparison of the comorbidity prevalence in BA and that in the general population is only roughly matched, as several parameters cannot be addressed e.g. the age group variance, gender difference, etc. BA-CNVs represent the large CNVs that were found in BA only against a large reference set, yet small CNVs intersect important genes can have been neglected while the platform variance between BA and the reference data set could have invited false discoveries. Last but not least, this is a rare complex disease study on a single population and is limited to the number of patients with the complete medical record only, we expect that replication survey in additional populations on both the phenotype and genotype findings can complete the story.

## Conclusions

Reconciling the findings at genotypic and phenotypic levels, our results suggest that a molecular network of rare and common genetic variants converge biologically in an inflammatory pathway that underpins the BA pathogenesis and drives the comorbidity of BA with immunity disorders. Moreover, from the patient complexity phenomenon and also the likely association of BA with immunity disease, we anticipate that a disease-disease network can exist for BA, which can help decode the BA phenotype from its location in the network while our knowledge of the liver development and immunobiology is still being updated. Yet indeed, putting this rare disease into a disease community requires a multi-centre research network to overcome the challenges posed by the analysis of small sample sizes.

## Additional files

Additional file 1: Supplementary methods and figures. (DOC 117 kb)

Additional file 2: Supplementary tables. (DOC 541 kb)

## Abbreviations

BA: Biliary Atresia
BA-CNV: Biliary Atresia private Copy Number Variant
CNV: Copy Number Variant
DGV: Database of Genomic Variants
eQTL: Expression Quantitative Trait Loci
FDR: False Discovery Rate
G6PD: Glucose-6-Phosphate Dehydrogenase
GPC: Genotype/gene Phenotype Correlation
IQR: Interquartile Range
LT: Liver Transplantation
PCR: Polymerase Chain Reaction
PPI: Protein-Protein Interaction
QC: Quality Control
SNP: Single Nucleotide Polymorphisms
