# Supplementary material for: Patient complexity and genotype-phenotype correlations in biliary atresia: a cross-sectional analysis
Source: BMC Med Genomics. 2017 Apr 17;10:22. doi: 10.1186/s12920-017-0259-0 (PMC5392958; doi:10.1186/s12920-017-0259-0)
Supplement: Supplementary file 1 — Supplementary methods and figures. (DOC 117 kb) [file 12920_2017_259_MOESM1_ESM.doc]

Supplementary materials for: Patients complexity and genotype-phenotype correlations in biliary atresia

**AUTHORS:** Guo CHENG^1^, Patrick Ho-Yu CHUNG^1^, Wai-kiu TANG^1^, Edwin Kin-Wai CHAN^6^, Man-Ting SO^1^, Pak-Chung SHAM^2,3,4,5^, Stacey S CHERNY^2,3,5^, Paul Kwong-Hang TAM^1,4^, Maria-Mercè GARCIA-BARCELÓ^1,3,4#^

**AFFILIATIONS:** ^1^Department of Surgery, ^2^Department of Psychiatry, ^3^Center for Genomic Sciences and ^4^Centre for Reproduction, Development, and Growth of the Li Ka Shing Faculty of Medicine, and, ^5^State Key Laboratory of Brain and Cognitive Sciences, The University of Hong Kong, Hong Kong SAR, China; ^6^Department of Surgery, the Chinese University of Hong Kong, Hong Kong SAR, China

# Supplementary methods

**CNV Calling From The Affymetrix 5.0 Array**

First Raw intensities of the probes were quantile-normalized across samples typed on the same plate using Affymetrix Power Tools (APT). Intensity measurements from all autosomal SNP probes and non-polymorphic copy number (CN) probes were used to identify deletions and duplications based on 3 software, Birdseye[**^1^**](#_ENREF_1) (of Birdsuite v1.5.5 package), PennCNV[**^2^**](#_ENREF_2) and iPattern[**^3^**](#_ENREF_3). Then copy number segments were detected using Birdseye on the by-plate basis. For PennCNV and iPattern, CNV calling was based on the three canonical genotype clusters, and Log R ratio (LRR) and B allele frequency (BAF) then were formed by the normalized intensities.

On the basis of intensity variation and the number of CNVs called, we filtered out individuals (2 cases) having abnormally large variation in intensities at genome-wide level [>3SD (standard deviation) from mean], and further these had large LRR (Log R Ratio) SD (>0.26) for PennCNV calls (1 case), resulting in 86 BA cases for downstream analysis. Further, to obtain high-confidence calls, we only used the consensus callings of CNVs called by PennCNV, Birdseye, and iPattern. Before selecting the consensus calls, quality control was done separately for CNVs predicted by three programs. As the CNV calling programs may artificially split the CNVs into multiple shorter CNVs, we filled in the gaps between calls if the gap were shorter than 20% of the total length of two adjacent CNVs.

To minimize false discoveries, only large CNVs called by all three algorithms were considered in the output. CNVs≧100kb that were called by at least 5 probes were considered. As the centromeric and telomeric regions are likely to harbour spurious CNV calls we removed any CNV segment with >50% overlap with these regions.

The post-calling steps are illustrated in Figure S1.

**Validation of CNV calls**

Copy number validation was performed by quantitative real-time Polymerase Chain Reaction (PCR) (ABI Prism 7900 Sequence Detection System; Applied Biosystems) using TaqMan® Copy Number Assay. The assay was carried out in quadruplicate with the TaqMan® Copy Number Reference Assay according to the manufacturer's protocol. The reference assay targets a copy-number neural region of RNaseP gene, serving as an internal standard. In brief, the mean differences in cycle threshold (ΔCT) between the testing gene and the reference probes for all replicates were computed and were subsequently normalized for copy number prediction. All probes cover the exonic region of genes.

**Gene-Based Genome Wide Association Study**

Gene-based association analysis that congregate effect of multiple SNPs within each genetic locus to elucidate BA-associated genes. Gene-based association analysis was carried out on 181 cases and 481 controls after quality control manners were take on the genotype and individual DNA performance as described. KGG gene set based association analysis was employed for the test. In details of the analysis, a gene locus was defined as the genomic coordinate of RefSeq gene boundary extending both 50kb upstream and downstream, and SNPs lying within a gene locus would be assumed to tag the variations of the gene function/expression, whereby BA-associations of those SNPs were congregated and translated as association of the gene with BA.

Besides, liver and lymphoblastoids expression quantitative trait loci (eQTL) in the GETX database were extracted to highlight genes with eQTL associated with BA. Those genes whose eQTL SNPs were associated with BA at the genome-wide significance level were consider to be associated with BA regardless of the association status in gene-based association test. In this step of analysis, the direct comparison of BA-association SNPs and the live/lymphoblastoids eQTL SNPs was facilitated by a whole-genome imputation procedure. Briefly, we chose the 1000 Genome Project genotype data (Phase I release on 2011, November) as reference panel[^4^](#_ENREF_4), which includes genotypes of 1092 individuals, and imputation was performed by IMPUTE2[^5^](#_ENREF_5).

# Supplementary Figures

| **Figure S1. Post-calling processing of CNVs** in order to get the BA CNVs.   |  | | --- | |  |   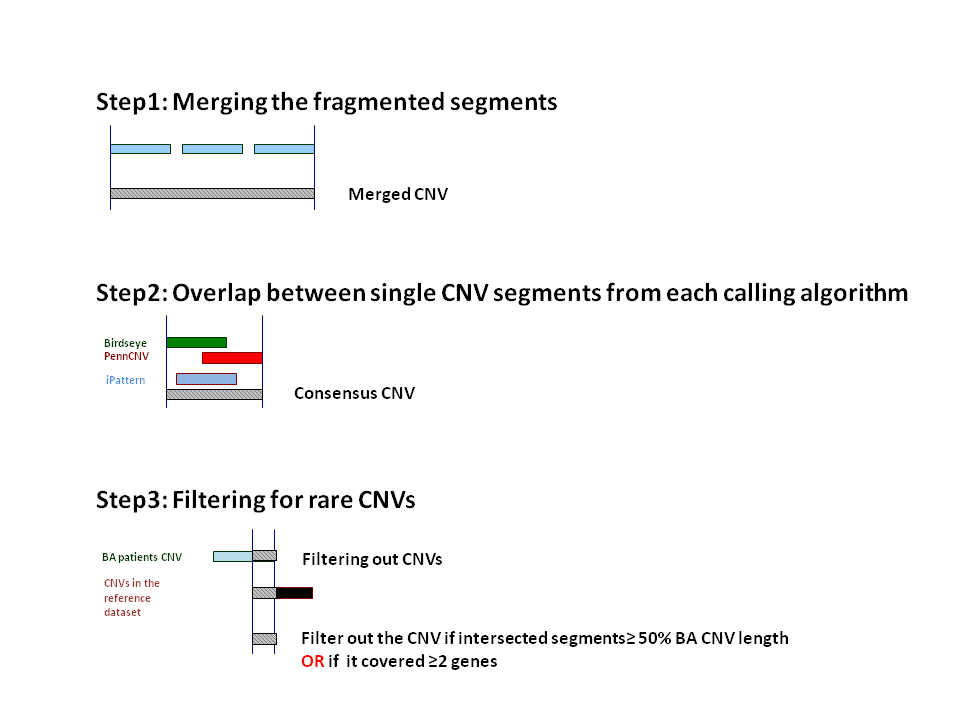 |
| --- | --- | --- |
|  |
|  |


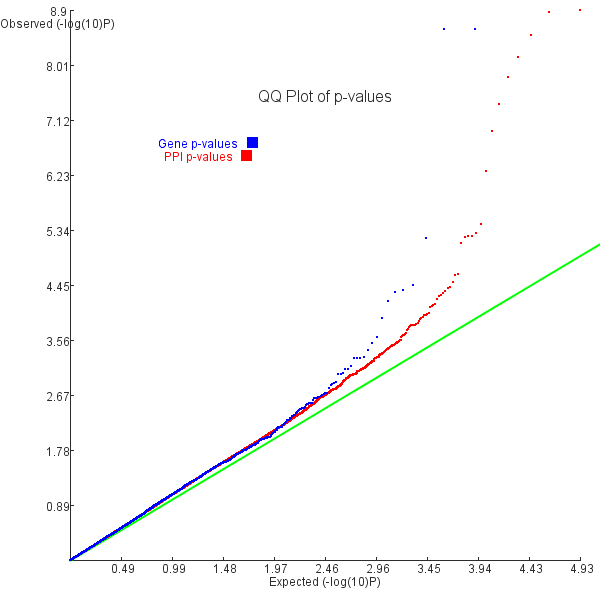


F**igure S2. QQ plot of the gene- (blue dots) and PPI- based (red dots) association *p* value**. Deviation from the expected distribution of a random set of p values (green line) suggests the existence of multiple disease association genes/networks under the multiple testing frame work.

# References

1. Korn JM, Kuruvilla FG, McCarroll SA, et al. Integrated genotype calling and association analysis of SNPs, common copy number polymorphisms and rare CNVs. *Nature genetics* 2008; **40**(10): 1253-60.

2. Wang K, Li M, Hadley D, et al. PennCNV: an integrated hidden Markov model designed for high-resolution copy number variation detection in whole-genome SNP genotyping data. *Genome Res* 2007; **17**(11): 1665-74.

3. Pinto D, Darvishi K, Shi X, et al. Comprehensive assessment of array-based platforms and calling algorithms for detection of copy number variants. *Nature biotechnology* 2011; **29**(6): 512-20.

4. Abecasis GR, Auton A, Brooks LD, et al. An integrated map of genetic variation from 1,092 human genomes. *Nature* 2012; **491**(7422): 56-65.

5. Marchini J, Howie B, Myers S, McVean G, Donnelly P. A new multipoint method for genome-wide association studies by imputation of genotypes. *Nature genetics* 2007; **39**(7): 906-13.
