# Supplementary material for: Patient complexity and genotype-phenotype correlations in biliary atresia: a cross-sectional analysis
Source: BMC Med Genomics. 2017 Apr 17;10:22. doi: 10.1186/s12920-017-0259-0 (PMC5392958; doi:10.1186/s12920-017-0259-0)
Supplement: Supplementary file 2 — Supplementary tables. (DOC 541 kb) [file 12920_2017_259_MOESM2_ESM.doc]

| **Table S1. ‘BA-CNVs’ and intersected genes** | | | | |  |
| --- | --- | --- | --- | --- | --- |
| **PID(non-BA diseases)** | **CNV Regions** | **Intersected Genes** | **Human Disease Gene (diseases)** | **Mouse* Immunity-related phenotype Gene** | **Mouse hepatobiliary-related phenotype Gene** |
| BA229(nil) | chr1:74089793-74709639(dup) | *FPGT,FPGT-TNNI3K,LRRIQ3,TNNI3K* | *Nil* | *Nil* | *Nil* |
| BA101(nil) | chr1:153410347-153634058(del) | *CHTOP,ILF2,NPR1,S100A1,S100A13,*  *S100A14,S100A16,S100A2 ,S100A3,*  *S100A4,S100A5 ,S100A6,S100A7,S100A7A,*  *S100A7L2,SNAPIN* | *NPR1*： cardiovascular/hypertension | *ILF2/NPR1/S100A4* | *Nil* |
| BA51(nil) | chr2:179178385-179296481(dup) | *DFNB59,OSBPL6,PRKRA* | *DFNB59:* Deafness, autosomal recessive 59 [MIM 610220] | *PRKRA* | *Nil* |
|  |  |  | *PRKRA:* Dystonia 16 [MIM612067] |  |  |
| BA144(nil) | chr3:107937857-108366960(dup) | *DZIP3,HHLA2,IFT57,KIAA1524,MYH15* | *MYH15:* cardiovascular phenotype | *Nil* | *Nil* |
| BA127(obstructive sleep apnea/ α thalassemia trait) | chr4:185748806-185965226(dup) | *ACSL1,HELT* | *Nil* | *Nil* | *Nil* |
| BA145(nil) | chr5:35702091-35969360(del) | *CAPSL,IL7R,SPEF2,UGT3A1* | *IL7R:*  severe combined immunodeficiency [MIM608971] | *IL7R/SPEF2* | *Nil* |
| BA60(deviated nasal septum and hypertrophy of nasal turbinates) | chr5:53263304-53820267(dup) | *ARL15,HSPB3,SNX18* | *HSPB3*: Neuronopathy, distal hereditary motor, type IIC [MIM613376] ^1^ | *Nil* | *Nil* |
| BA52(nil) | chr5:114339255-115373181(dup) | *AP3S1,AQPEP,ATG12,CCDC112,CDO1,*  *FEM1C, LOC644100,PGGT1B,TICAM2,*  *TMED7,TMED7-TICAM2,TRIM36* | *Nil* | *AP3S1/ATG12* | *Nil* |
| BA99(diabetes mellitus type 1) | chr6:7167068-7265067(dup) | *RREB1,SSR1* | *Nil* | *Nil* | *Nil* |
| BA80(jejunal atresia) | chr6:25283033-25730027(dup) | *HIST1H2AA,HIST1H2BA,LRRC16A,*  *SCGN,SLC17A4* | *Nil* | *Nil* | *Nil* |
| BA47(Intestinal malrotation) | chr6:37625389-37727046(del) | *MDGA1* | *Nil* | *Nil* | *Nil* |
| BA98(nil) | chr6:38860093-39439484(dup) | *C6orf64,DNAH8,GLP1R,KCNK16,KCNK17*  *,KCNK5,KIF6* | *GLP1R:* metabolic diseases^2^ | *Nil* | *Nil* |
| BA116(nil) | chr6:50365637-50639886(del) | *TFAP2D* | *Nil* | *Nil* | *Nil* |
| BA60(deviated nasal septum and hypertrophy of nasal turbinates) | chr6:101975319-102443447(dup) | *GRIK2* | *GRIK2:* mental retardation, autosomal recessive, 6 [MIM611092]; asthma^3^ | *Nil* | *Nil* |
| BA35(G6PD deficiency, epilepsy ) | chr6:127976130-128093924(dup) | *THEMIS* | *Nil* | *THEMIS* | *Nil* |
| BA135(nil) | chr7:10865554-11567370(dup) | *NDUFA4,PHF14,THSD7A* | *NDUFA4*: Dandy-Walker syndrome [MIM220200]; neurological diseases^4,5^ | *Nil* | *Nil* |
| BA64(hyperlipidemia) | chr7:10865554-11568669(dup) |  | *THSD7A*: familial thrombosis / osteoporosis^6,7^ |  |  |
| BA52(nil) | chr8:8528859-8625511(del) | *CLDN23,MFHAS1* | *MFHAS1:* Malignant fibrous histiocytoma | *Nil* | *Nil* |
| BA228(nil) | chr13:37812597-38163991(dup) | *POSTN* | *Nil* | *POSTN* | *Nil* |
| BA119(nil) | chr16:73797008-74708318(dup) | *CLEC18B,GLG1,MLKL,PSMD7,RFWD3* | *PSMD7*: ankylosing spondylitis; prostate cancer ^8,9^ | *PSMD7* | *Nil* |
| BA99(diabetes mellitus type 1) | chr16:81947921-82095870(dup) | *HSD17B2,PLCG2,SDR42E1* | *HSD17B2*: malignancy; estrogen/androgen related diseases ^10-12^ | *PLCG2* | *Nil* |
|  |  |  | *PLCG2*: PLCG2-associated antibody deficiency and immune dysregulation; diabetes mellitus type 1^13^ |  |  |
| BA44(hashimoto thyroiditis/palmar hyperhydrosis) | chr18:7080664-7584939(dup) | *LAMA1,LRRC30,PTPRM* | *LAMA1:*diabetes mellitus; myopia^14^ ^15^ | *Nil* | *Nil* |
|  |  |  | *PTPRM:* autoimmune disease (systemic lupus erythematosus,multiple sclerosi ^16,17^) |  |  |
| BA50(nil) | chr18:11452145-11810830(dup) | *GNAL* | *Nil* | *Nil* | *Nil* |
| BA68(eczema ) | chr18:55546006-56018494(dup) | *NEDD4L* | *NEDD4L:*asthma; metabolism; hypertension disease ^18,19^ | *Nil* | *Nil* |
| BA46(congenital hypothyroidism) | chr20:983957-1126856(dup) | *PSMF1,RSPO4* | *RSPO4:congenital hyponychia^20^* | *Nil* | *Nil* |
| BA123(nil) | chr20:10533666-10694525(del) | *JAG1,SLX4IP* | *JAG1: Alagille syndrome[OMIM118450]; BA^21^* | *Nil* | *Nil* |
| BA79(nil) | chr20:20371406-20684642(dup) | *INSM1,RALGAPA2* | *Nil* | *Nil* | *Nil* |
| BA98(nil) | chr20:58489614-59475881(dup) | *C20orf177,C20orf197,CDH26,PPP1R3D,SYCP2* | *Nil* | *Nil* | *PPP1R3D* |
| BA304(right vesicoureteric reflux grade IV) | chr22:44654613-45177704(dup) | *ARHGAP8,KIAA1644,LDOC1L,PRR5,PRR5-ARHGAP8* | *Nil* | *Nil* | *Nil* |
| *according to the MGI database (http://www.informatics.jax.org/) | | | | | |

| **Table S2. BA-associated genes through genebased genome wide association analysis.** | | | | | | | |
| --- | --- | --- | --- | --- | --- | --- | --- |
| **Gene_Symbol** | **Gene_*P* Value** | **Chr** | **Gene-Position** | **SNP** | **SNP-Position** | **Gene_Feature** | ***P*** |
| PAOX | 1.86E-12 | 10 | 135192740-135205200 | rs11101722 | 135190428 | upstream | 6.10E-13 |
| PI4KA | 6.53E-09 | 22 | 21061978-21213100 | rs9612574 | 21011215 | upstream | 1.22E-09 |
| TMEM191A | 6.65E-09 | 22 | 21055401-21058891 | rs9612574 | 21011215 | upstream | 1.22E-09 |
| POM121L4P | 9.02E-09 | 22 | 21043842-21046009 | rs9612574 | 21011215 | upstream | 1.22E-09 |
| UBE3C | 9.50E-09 | 7 | 156931654-157062066 | rs6966038 | 156880397 | intergenic | 3.75E-09 |
| MED15 | 1.83E-08 | 22 | 20861885-20941919 | rs9612574 | 21011215 | upstream | 1.22E-09 |
|  | - | - | - | rs1110462 | 20881384 | intronic | 0.0201 |
| RBFOX1 | 2.41E-08 | 16 | 6069131-7763340 | rs17139085 | 6050072 | upstream | 1.14E-10 |
|  | - | - | - | rs11647023 | 6963769 | intronic | 0.000214 |
|  | - | - | - | rs17143361 | 7355257 | intronic | 0.00043 |
|  | - | - | - | rs9929481 | 6043105 | upstream | 0.000489 |
|  | - | - | - | rs9925034 | 6578168 | intronic | 0.002205 |
|  | - | - | - | rs1492374 | 6909943 | intronic | 0.009464 |
|  | - | - | - | rs6500880 | 6978473 | intronic | 0.009607 |
|  | - | - | - | rs7191914 | 7333464 | intronic | 0.009809 |
|  | - | - | - | rs9928304 | 6978845 | intronic | 0.0109 |
|  | - | - | - | rs1388807 | 6925780 | intronic | 0.01182 |
|  | - | - | - | rs7190863 | 7333350 | intronic | 0.01188 |
|  | - | - | - | rs11865410 | 7333565 | intronic | 0.01344 |
|  | - | - | - | rs7500396 | 6903126 | intronic | 0.01389 |
|  | - | - | - | rs7196563 | 7333407 | intronic | 0.0157 |
|  | - | - | - | rs17235672 | 6930541 | intronic | 0.01908 |
|  | - | - | - | rs7185031 | 6930913 | intronic | 0.02066 |
|  | - | - | - | rs12932136 | 7611972 | intronic | 0.02152 |
|  | - | - | - | rs1507015 | 7571291 | intronic | 0.02229 |
|  | - | - | - | rs2063090 | 7571354 | intronic | 0.02229 |
|  | - | - | - | rs7191957 | 6930927 | intronic | 0.02231 |
|  | - | - | - | rs11077168 | 7342776 | intronic | 0.02318 |
|  | - | - | - | rs11077017 | 6284886 | intronic | 0.02576 |
|  | - | - | - | rs7204136 | 6981821 | intronic | 0.02714 |
|  | - | - | - | rs17826831 | 6928220 | intronic | 0.02934 |
|  | - | - | - | rs1882586 | 7329806 | intronic | 0.03317 |
|  | - | - | - | rs6500983 | 7605887 | intronic | 0.03621 |
|  | - | - | - | rs17737424 | 7200593 | intronic | 0.03824 |
|  | - | - | - | rs7197461 | 6158260 | intronic | 0.03887 |
|  | - | - | - | rs8059779 | 7544552 | intronic | 0.03914 |
|  | - | - | - | rs2035247 | 7069055 | intronic | 0.04052 |
|  | - | - | - | rs12709151 | 6221275 | intronic | 0.04244 |
|  | - | - | - | rs6500981 | 7593574 | intronic | 0.04272 |
|  | - | - | - | rs2345485 | 6763215 | intronic | 0.04589 |
|  | - | - | - | rs12445513 | 6903080 | intronic | 0.04609 |
|  | - | - | - | rs7204945 | 7668395 | intronic | 0.04695 |
|  | - | - | - | rs4787050 | 7668999 | intronic | 0.04695 |
|  | - | - | - | rs4787048 | 7664101 | intronic | 0.04776 |
|  | - | - | - | rs2795573 | 6508612 | intronic | 0.04954 |
|  | - | - | - | rs3826208 | 7720019 | intronic | 0.04981 |
| LOC645249 | 2.96E-08 | 7 | 156803555-156804009 | rs6966038 | 156880397 | intergenic | 3.75E-09 |
|  | - | - | - | rs10262191 | 156804509 | upstream | 0.03006 |
| GAL | 3.02E-08 | 11 | 68451982-68458643 | rs2510389 | 68408003 | upstream | 4.52E-09 |
|  | - | - | - | rs882979 | 68408288 | upstream | 0.000155 |
|  | - | - | - | rs4073959 | 68481489 | intronic | 0.02405 |
|  | - | - | - | rs4930243 | 68499873 | intronic | 0.02655 |
| MNX1 | 4.53E-08 | 7 | 156797546-156809118 | rs6966038 | 156880397 | intergenic | 3.75E-09 |
|  | - | - | - | rs10262191 | 156804509 | upstream | 0.03006 |
| PPP6R3 | 5.14E-08 | 11 | 68228185-68382801 | rs2510389 | 68408003 | upstream | 4.52E-09 |
|  | - | - | - | rs882979 | 68408288 | upstream | 0.000155 |
| SNORA80B | 8.01E-08 | 2 | 10586839-10586975 | rs3755256 | 10566413 | intronic | 7.34E-09 |
|  | - | - | - | rs818154 | 10643636 | intergenic | 0.002722 |
|  | - | - | - | rs7591360 | 10616213 | upstream | 0.01341 |
| ODC1 | 8.01E-08 | 2 | 10580496-10588680 | rs3755256 | 10566413 | intronic | 7.34E-09 |
|  | - | - | - | rs818154 | 10643636 | intergenic | 0.002722 |
|  | - | - | - | rs7591360 | 10616213 | upstream | 0.01341 |
| MTL5 | 8.16E-08 | 11 | 68474907-68518988 | rs2510389 | 68408003 | upstream | 4.52E-09 |
|  | - | - | - | rs882979 | 68408288 | upstream | 0.000155 |
|  | - | - | - | rs3763831 | 68540315 | intronic | 0.003094 |
|  | - | - | - | rs4073959 | 68481489 | intronic | 0.02405 |
|  | - | - | - | rs4930243 | 68499873 | intronic | 0.02655 |
|  | - | - | - | rs2924683 | 68550303 | intronic | 0.03813 |
| SOX8 | 1.51E-07 | 16 | 1031807-1036979 | rs12595929 | 1007762 | intronic | 5.29E-08 |
| HPCAL1 | 2.09E-07 | 2 | 10443029-10567743 | rs3755256 | 10566413 | intronic | 7.34E-09 |
|  | - | - | - | rs818154 | 10643636 | intergenic | 0.002722 |
|  | - | - | - | rs7591360 | 10616213 | upstream | 0.01341 |
|  | - | - | - | rs6432089 | 10436397 | upstream | 0.02528 |
|  | - | - | - | rs4669564 | 10422341 | upstream | 0.02936 |
| SLC2A6 | 2.38E-07 | 9 | 136336215-136344276 | rs4962153 | 136323753 | intronic | 8.00E-08 |
| LMF1 | 2.56E-07 | 16 | 903634-1031318 | rs12595929 | 1007762 | intronic | 5.29E-08 |
| REXO4 | 3.12E-07 | 9 | 136271181-136283292 | rs4962153 | 136323753 | intronic | 8.00E-08 |
| CACFD1 | 3.55E-07 | 9 | 136325086-136335909 | rs4962153 | 136323753 | intronic | 8.00E-08 |
| C9orf96 | 3.91E-07 | 9 | 136243283-136271220 | rs4962153 | 136323753 | intronic | 8.00E-08 |
| ADAMTS13 | 4.00E-07 | 9 | 136279458-136324525 | rs4962153 | 136323753 | intronic | 8.00E-08 |
| DUX2 | 4.63E-07 | 4 | 190988992-190990372 | rs3813280 | 190724415 | upstream | 6.73E-08 |
| SURF4 | 4.66E-07 | 9 | 136228324-136244820 | rs4962153 | 136323753 | intronic | 8.00E-08 |
| TMEM8C | 4.98E-07 | 9 | 136379707-136390068 | rs4962153 | 136323753 | intronic | 8.00E-08 |
| ITPK1AS1 | 5.18E-07 | 14 | 93533796-93538478 | rs749619 | 93523893 | intronic | 6.13E-08 |
|  | - | - | - | rs7157141 | 93629958 | upstream | 0.03501 |
| ITPK1 | 6.91E-07 | 14 | 93403258-93582263 | rs749619 | 93523893 | intronic | 6.13E-08 |
|  | - | - | - | rs7157141 | 93629958 | upstream | 0.03501 |
| LOC100129027 | 8.20E-07 | 21 | 47247754-47256333 | rs3788239 | 47351172 | intronic | 1.02E-07 |
| COL6A1 | 9.46E-07 | 21 | 47401662-47424963 | rs3788239 | 47351172 | intronic | 1.02E-07 |
| PCBP3 | 2.06E-06 | 21 | 47269874-47362368 | rs3788239 | 47351172 | intronic | 1.02E-07 |
| CBFA2T3 | 3.27E-06 | 16 | 88941262-89043504 | rs7193497 | 89064018 | upstream | 4.60E-07 |
|  | - | - | - | rs6500514 | 89057560 | upstream | 0.01883 |
| TMEM236 | 5.51E-06 | 10 | 17794259-18089854 | rs17141517 | 17746300 | intronic | 1.22E-06 |
| ADAMTS17 | 8.57E-06 | 15 | 100511642-100882183 | rs2727217 | 100525940 | intronic | 1.14E-07 |
|  | - | - | - | rs8027190 | 100640203 | intronic | 0.01106 |
|  | - | - | - | rs11858287 | 100888385 | upstream | 0.01605 |
|  | - | - | - | rs2418488 | 100641890 | intronic | 0.02059 |
|  | - | - | - | rs7182422 | 100859568 | intronic | 0.02297 |
|  | - | - | - | rs873843 | 100510424 | downstream | 0.03874 |
|  | - | - | - | rs2573668 | 100541775 | intronic | 0.04184 |
| STAM | 9.29E-06 | 10 | 17683282-17758821 | rs17141517 | 17746300 | intronic | 1.22E-06 |
| TCP10 | 1.44E-05 | 6 | 167786576-167797998 | rs4546466 | 167838992 | upstream | 9.77E-07 |
| F13A1 | 1.56E-05 | 6 | 6144310-6320924 | rs13198304 | 6356481 | upstream | 2.04E-07 |
|  | - | - | - | rs2274394 | 6152210 | intronic | 0.005779 |
|  | - | - | - | rs9504681 | 6138153 | downstream | 0.005779 |
|  | - | - | - | rs13194966 | 6150988 | intronic | 0.005979 |
|  | - | - | - | rs6913691 | 6153486 | intronic | 0.005979 |
|  | - | - | - | rs4527760 | 6078202 | intergenic | 0.006912 |
|  | - | - | - | rs6914953 | 6153442 | intronic | 0.007132 |
|  | - | - | - | rs3799563 | 6150737 | intronic | 0.007555 |
|  | - | - | - | rs35010441 | 6155136 | intronic | 0.007697 |
|  | - | - | - | rs13203312 | 6153099 | intronic | 0.007977 |
|  | - | - | - | rs6937073 | 6153378 | intronic | 0.008094 |
|  | - | - | - | rs1351656 | 6131323 | downstream | 0.008873 |
|  | - | - | - | rs1998878 | 6082161 | intergenic | 0.009924 |
|  | - | - | - | rs6597185 | 6076432 | intergenic | 0.01257 |
|  | - | - | - | rs17141840 | 6173369 | intronic | 0.0151 |
|  | - | - | - | rs3799564 | 6146492 | intronic | 0.02548 |
|  | - | - | - | rs6597193 | 6140095 | downstream | 0.02595 |
|  | - | - | - | rs3886388 | 6365980 | upstream | 0.02876 |
|  | - | - | - | rs9392772 | 6363272 | upstream | 0.03001 |
|  | - | - | - | rs911509 | 6358199 | upstream | 0.03187 |
|  | - | - | - | rs11757413 | 6357893 | upstream | 0.03187 |
|  | - | - | - | rs11754511 | 6356212 | upstream | 0.03187 |
|  | - | - | - | rs3851513 | 6274160 | intronic | 0.03708 |
|  | - | - | - | rs4960172 | 6161661 | intronic | 0.03932 |
|  | - | - | - | rs3778360 | 6150364 | intronic | 0.04513 |
|  | - | - | - | rs9392745 | 6084558 | intergenic | 0.04756 |
|  | - | - | - | rs4142290 | 6154606 | intronic | 0.04888 |
| MTHFD1L | 1.69E-05 | 6 | 151186814-151423023 | rs17427019 | 151156166 | intronic | 5.79E-07 |
|  | - | - | - | rs487637 | 151339441 | intronic | 0.03594 |
| XPNPEP1 | 1.79E-05 | 10 | 111624523-111683311 | rs12571674 | 111717607 | upstream | 9.72E-06 |
|  | - | - | - | rs731793 | 111716666 | upstream | 1.71E-05 |
|  | - | - | - | rs11194926 | 111700921 | upstream | 1.85E-05 |
|  | - | - | - | rs734165 | 111697788 | upstream | 2.15E-05 |
|  | - | - | - | rs921351 | 111704714 | upstream | 2.32E-05 |
|  | - | - | - | rs7080576 | 111710816 | upstream | 2.60E-05 |
|  | - | - | - | rs944966 | 111658333 | intronic | 0.001725 |
|  | - | - | - | rs1973972 | 111651132 | intronic | 0.001836 |
|  | - | - | - | rs7075964 | 111631264 | intronic | 0.02102 |
|  | - | - | - | rs2274978 | 111631664 | intronic | 0.02918 |
|  | - | - | - | rs12768489 | 111709877 | upstream | 0.03102 |
|  | - | - | - | rs11194894 | 111612665 | downstream | 0.04712 |
| MAMDC2 | 1.81E-05 | 9 | 72658496-72841888 | rs2871297 | 72813680 | intronic | 7.70E-06 |
| TTLL2 | 2.09E-05 | 6 | 167738573-167756177 | rs4546466 | 167838992 | upstream | 9.77E-07 |
| NPHS2 | 2.16E-05 | 1 | 179519673-179545084 | rs4652421 | 179563716 | intronic | 2.19E-06 |
| PLEKHG1 | 2.29E-05 | 6 | 150920998-151164799 | rs17427019 | 151156166 | intronic | 5.79E-07 |
| OR7E156P | 2.63E-05 | 13 | 64311567-64316701 | rs9571013 | 64303506 | upstream | 2.63E-05 |
| TMEM117 | 2.74E-05 | 12 | 44229663-44783545 | rs1492310 | 44549434 | intronic | 1.31E-06 |
|  | - | - | - | rs4459374 | 44551066 | intronic | 0.02255 |
|  | - | - | - | rs17094231 | 44602375 | intronic | 0.04211 |
|  | - | - | - | rs10785472 | 44312773 | intronic | 0.04557 |
|  | - | - | - | rs7953173 | 44488755 | intronic | 0.0473 |
|  | - | - | - | rs2407790 | 44431492 | intronic | 0.04861 |
| LOC100505933 | 3.19E-05 | 10 | 11705317-111765839 | rs17095355 | 111735749 | upstream | 7.35E-06 |
|  | - | - | - | rs12571674 | 111717607 | upstream | 9.72E-06 |
|  | - | - | - | rs731793 | 111716666 | upstream | 1.71E-05 |
|  | - | - | - | rs11194926 | 111700921 | upstream | 1.85E-05 |
|  | - | - | - | rs734165 | 111697788 | upstream | 2.15E-05 |
|  | - | - | - | rs921351 | 111704714 | upstream | 2.32E-05 |
|  | - | - | - | rs7080576 | 111710816 | upstream | 2.60E-05 |
|  | - | - | - | rs3862006 | 111750763 | upstream | 7.41E-05 |
|  | - | - | - | rs944966 | 111658333 | intronic | 0.001725 |
|  | - | - | - | rs12768489 | 111709877 | upstream | 0.03102 |
| LY86AS1 | 3.54E-05 | 6 | 6346697-6623059 | rs13198304 | 6356481 | upstream | 2.04E-07 |
|  | - | - | - | rs3886388 | 6365980 | upstream | 0.02876 |
|  | - | - | - | rs9392772 | 6363272 | upstream | 0.03001 |
|  | - | - | - | rs9328360 | 6400148 | ncRNA | 0.03073 |
|  | - | - | - | rs911509 | 6358199 | upstream | 0.03187 |
|  | - | - | - | rs11757413 | 6357893 | upstream | 0.03187 |
|  | - | - | - | rs11754511 | 6356212 | upstream | 0.03187 |
|  | - | - | - | rs12196470 | 6389847 | ncRNA | 0.04133 |
|  | - | - | - | rs4543420 | 6384147 | ncRNA | 0.04966 |
| MIR644A | 3.74E-05 | 20 | 33054129-33054223 | rs6088515 | 33110041 | intronic | 8.31E-06 |
|  | - | - | - | rs6059827 | 33016357 | intronic | 0.001269 |
|  | - | - | - | rs6059867 | 33078102 | intronic | 0.001269 |
|  | - | - | - | rs6059866 | 33075809 | intronic | 0.002728 |
|  | - | - | - | rs6088519 | 33132190 | upstream | 0.00615 |
| UBE2E1 | 4.06E-05 | 3 | 23847383-23933131 | rs7634023 | 23843472 | upstream | 8.88E-06 |
|  | - | - | - | rs2168424 | 23869247 | intronic | 0.000382 |
|  | - | - | - | rs6550801 | 23843619 | upstream | 0.01251 |
|  | - | - | - | rs6774787 | 23859060 | intronic | 0.04666 |
| AXDND1 | 4.07E-05 | 1 | 179334854-179523870 | rs4652421 | 179563716 | intronic | 2.19E-06 |
|  | - | - | - | rs4652421 | 179563716 | intronic | 2.19E-06 |
| ADD3 | 4.12E-05 | 10 | 111765725-111895323 | rs17095355 | 111735749 | upstream | 7.35E-06 |
|  | - | - | - | rs12571674 | 111717607 | upstream | 9.72E-06 |
|  | - | - | - | rs731793 | 111716666 | upstream | 1.71E-05 |
|  | - | - | - | rs3862006 | 111750763 | upstream | 7.41E-05 |
|  | - | - | - | rs12243027 | 111924816 | upstream | 8.96E-05 |
|  | - | - | - | rs2501577 | 111846686 | intronic | 0.000111 |
|  | - | - | - | rs11194981 | 111861053 | intronic | 0.000163 |
|  | - | - | - | rs6584970 | 111847819 | intronic | 0.000181 |
|  | - | - | - | rs2501575 | 111849863 | intronic | 0.000193 |
|  | - | - | - | rs4244276 | 111935177 | upstream | 0.000761 |
| CBR4 | 4.80E-05 | 4 | 169908741-169931468 | rs6843588 | 169899959 | downstream | 1.56E-05 |
|  | - | - | - | rs1458292 | 169935880 | upstream | 0.04007 |
| LOC100507299 | 4.88E-05 | 9 | 72808914-72873782 | rs2871297 | 72813680 | intronic | 7.70E-06 |
| DYNLRB1 | 4.98E-05 | 20 | 33104188-33128762 | rs6088515 | 33110041 | intronic | 8.31E-06 |
|  | - | - | - | rs764597 | 33161224 | intronic | 0.000575 |
|  | - | - | - | rs6059867 | 33078102 | intronic | 0.001269 |
|  | - | - | - | rs6059866 | 33075809 | intronic | 0.002728 |
|  | - | - | - | rs6088519 | 33132190 | upstream | 0.00615 |
| MAP1LC3A | 5.23E-05 | 20 | 33134687-33148149 | rs6088515 | 33110041 | intronic | 8.31E-06 |
|  | - | - | - | rs764597 | 33161224 | intronic | 0.000575 |
|  | - | - | - | rs6059867 | 33078102 | intronic | 0.001269 |
|  | - | - | - | rs6059956 | 33220069 | intronic | 0.001446 |
|  | - | - | - | rs6059866 | 33075809 | intronic | 0.002728 |
|  | - | - | - | rs6088519 | 33132190 | upstream | 0.00615 |
| LOC100507244 | 5.88E-05 | 9 | 72768050-72790804 | rs2871297 | 72813680 | intronic | 7.70E-06 |
| KIAA1107 | 6.35E-05 | 1 | 92632608-92650280 | rs4437922 | 92581817 | intronic | 6.35E-05 |
| SMC5 | 6.58E-05 | 9 | 72873877-72969789 | rs2871297 | 72813680 | intronic | 7.70E-06 |
| LINC00297 | 6.72E-05 | 13 | 30446749-30462542 | rs9314986 | 30458736 | upstream | 3.26E-06 |
|  | - | - | - | rs1590501 | 30456306 | upstream | 1.59E-05 |
|  | - | - | - | rs9551767 | 30462711 | upstream | 0.008388 |
|  | - | - | - | rs9805864 | 30455344 | upstream | 0.01431 |
|  | - | - | - | rs9578147 | 30464034 | upstream | 0.01762 |
| NLRP5 | 7.54E-05 | 19 | 56511091-56573174 | rs3097879 | 56528045 | intronic | 1.08E-05 |
|  | - | - | - | rs16986877 | 56521380 | intronic | 0.02311 |
| ITCH | 7.55E-05 | 20 | 32951040-33099198 | rs6088515 | 33110041 | intronic | 8.31E-06 |
|  | - | - | - | rs764597 | 33161224 | intronic | 0.000575 |
|  | - | - | - | rs6059827 | 33016357 | intronic | 0.001269 |
|  | - | - | - | rs6059867 | 33078102 | intronic | 0.001269 |
|  | - | - | - | rs6087577 | 32955422 | intronic | 0.001442 |
|  | - | - | - | rs4911420 | 32998653 | intronic | 0.002059 |
|  | - | - | - | rs6059866 | 33075809 | intronic | 0.002728 |
|  | - | - | - | rs6088519 | 33132190 | upstream | 0.00615 |
|  | - | - | - | rs6088466 | 32913533 | upstream | 0.01924 |
| TDRD5 | 7.69E-05 | 1 | 179560747-179660407 | rs4652421 | 179563716 | intronic | 2.19E-06 |
| MIR3650 | 8.22E-05 | 5 | 38557603-38557663 | rs1444884 | 38615655 | upstream | 1.12E-05 |
|  | - | - | - | rs17427485 | 38569856 | intronic | 0.01463 |
|  | - | - | - | rs327276 | 38633263 | upstream | 0.01619 |
|  | - | - | - | rs3797165 | 38524824 | intronic | 0.04035 |
| PIGU | 0.000107 | 20 | 33148345-33265089 | rs6088515 | 33110041 | intronic | 8.31E-06 |
|  | - | - | - | rs764597 | 33161224 | intronic | 0.000575 |
|  | - | - | - | rs6059956 | 33220069 | intronic | 0.001446 |
|  | - | - | - | rs6088519 | 33132190 | upstream | 0.00615 |
|  | - | - | - | rs6060001 | 33294352 | intronic | 0.00647 |
| UBL3 | 0.000113 | 13 | 30338544-30424820 | rs9314986 | 30458736 | upstream | 3.26E-06 |
|  | - | - | - | rs1590501 | 30456306 | upstream | 1.59E-05 |
|  | - | - | - | rs9551767 | 30462711 | upstream | 0.008388 |
|  | - | - | - | rs4769769 | 30291226 | downstream | 0.009196 |
|  | - | - | - | rs9805864 | 30455344 | upstream | 0.01431 |
|  | - | - | - | rs9578147 | 30464034 | upstream | 0.01762 |
|  | - | - | - | rs3818368 | 30351265 | intronic | 0.02237 |
| BTBD8 | 0.000126 | 1 | 92545861-92613401 | rs4437922 | 92581817 | intronic | 6.35E-05 |
| KRAS | 0.000131 | 12 | 25357722-25403865 | rs6487465 | 25410282 | upstream | 4.91E-05 |
|  | - | - | - | rs7312175 | 25404603 | upstream | 0.02879 |
|  | - | - | - | rs4559767 | 25298386 | intronic | 0.03181 |
| LYRM5 | 0.000131 | 12 | 25348149-25357949 | rs6487465 | 25410282 | upstream | 4.91E-05 |
|  | - | - | - | rs7312175 | 25404603 | upstream | 0.02879 |
|  | - | - | - | rs4559767 | 25298386 | intronic | 0.03181 |

| **Table S3. Genes with eQTL intersect SNPs of suggestive BA-association p values** | | | | | |
| --- | --- | --- | --- | --- | --- |
| **Genes** | **Probe Loc** | **QTL SNP** | **SNP Loc** | **Tissue** | **BA association *p* of SNP** |
| *RHCE* | chr1:25688740 | rs2821096 | chr1:47742726 | liver | 0.002934 |
| *B4GALT2* | chr1:44456678 | rs2788371 | chr1:44343962 | lymphoblastoid | 0.004612 |
|  |  | rs3791041 | chr1:44202991 | lymphoblastoid | 0.004382 |
|  |  | rs4660260 | chr1:44195353 | lymphoblastoid | 0.004088 |
|  |  | rs4660261 | chr1:44229597 | lymphoblastoid | 0.003153 |
|  |  | rs6429638 | chr1:44211010 | lymphoblastoid | 0.003163 |
|  |  | rs803675 | chr1:44353237 | lymphoblastoid | 0.0003659 |
|  |  | rs803679 | chr1:44349405 | lymphoblastoid | 0.0003649 |
|  |  | rs917294 | chr1:44249485 | lymphoblastoid | 0.0003257 |
| *S100A13* | chr1:153598851 | rs913861 | chr1:153622220 | lymphoblastoid | 0.003078 |
| *SMG5* | chr1:156219015 | rs933489 | chr1:156235120 | liver | 0.00267 |
| *C1orf115* | chr1:220872181 | rs337154 | chr1:220871123 | lymphoblastoid | 0.0008821 |
| *CEBPZ* | chr2:37450313 | rs1348748 | chr2:38229418 | lymphoblastoid | 0.0004272 |
|  |  | rs1446297 | chr2:38208233 | lymphoblastoid | 0.00108 |
|  |  | rs1992735 | chr2:38223061 | lymphoblastoid | 0.0005473 |
|  |  | rs2373327 | chr2:38222086 | lymphoblastoid | 0.001473 |
|  |  | rs2373329 | chr2:38227561 | lymphoblastoid | 0.0004477 |
| *CEBPZ* | chr2:37454687 | rs2373329 | chr2:38227561 | lymphoblastoid | 0.0004477 |
| *C2orf74* | chr2:61390265 | rs778139 | chr2:61684564 | lymphoblastoid | 0.002533 |
|  |  | rs967968 | chr2:61656056 | lymphoblastoid | 0.004439 |
| *LOC151174* | chr2:239133854 | rs3739064 | chr2:239176386 | lymphoblastoid | 0.0002549 |
| *LCORL* | chr4:17844843 | rs10261505 | chr7:154498135 | liver | 0.0002249 |
| *AGGF1* | chr5:76326232 | rs10261505 | chr7:154498135 | liver | 0.0002249 |
| *MSH3* | chr5:79950294 | rs6151896 | chr5:80121646 | liver | 0.002019 |
| *P4HA2* | chr5:131527531 | rs40401 | chr5:131396478 | lymphoblastoid | 0.00411 |
|  | chr6:26370550 | rs4634439 | chr6:26598004 | lymphoblastoid | 0.003318 |
|  | chr6:26373125 | rs4634439 | chr6:26598004 | lymphoblastoid | 0.003318 |
|  |  | rs4634439 | chr6:26598004 | lymphoblastoid | 0.003318 |
|  | chr6:26376757 | rs4634439 | chr6:26598004 | lymphoblastoid | 0.003318 |
| *C7orf46* | chr7:23731174 | rs6974500 | chr7:23744549 | lymphoblastoid | 0.003916 |
| *STK31* | chr7:23749891 | rs6974500 | chr7:23744549 | liver | 0.003916 |
| *RABGEF1* | chr7:66275925 | rs10247591 | chr7:66358094 | lymphoblastoid | 0.00373 |
|  |  | rs10267922 | chr7:66345205 | lymphoblastoid | 0.002516 |
|  |  | rs10275956 | chr7:66251773 | lymphoblastoid | 0.004953 |
|  |  | rs12537474 | chr7:66305022 | lymphoblastoid | 0.002181 |
|  |  | rs2077180 | chr7:66265445 | lymphoblastoid | 0.003317 |
| *tcag7.967* | chr7:66275925 | rs10247591 | chr7:66358094 | lymphoblastoid | 0.00373 |
|  |  | rs10267922 | chr7:66345205 | lymphoblastoid | 0.002516 |
|  |  | rs10275956 | chr7:66251773 | lymphoblastoid | 0.004953 |
|  |  | rs12537474 | chr7:66305022 | lymphoblastoid | 0.002181 |
|  |  | rs2077180 | chr7:66265445 | lymphoblastoid | 0.003317 |
| *GNAI1* | chr7:79764140 | rs10261505 | chr7:154498135 | liver | 0.0002249 |
| *C7orf49* | chr7:134850744 | rs292553 | chr7:134871348 | lymphoblastoid | 0.0003017 |
|  |  | rs292594 | chr7:134891308 | lymphoblastoid | 0.0004592 |
|  |  | rs292601 | chr7:134895532 | lymphoblastoid | 0.0007373 |
| *PAXIP1* | chr7:154735400 | rs10261505 | chr7:154498135 | liver | 0.0002249 |
| *FAM164A* | chr8:79627471 | rs6993386 | chr8:79654145 | lymphoblastoid | 0.004968 |
| *EIF3H* | chr8:117657055 | rs12675038 | chr8:117706218 | liver | 0.004797 |
| *CDK5RAP2* | chr9:123151259 | rs1547266 | chr9:123163476 | lymphoblastoid | 0.00422 |
|  |  | rs3780674 | chr9:123166919 | lymphoblastoid | 0.003533 |
| *USMG5* | chr10:105154020 | rs11191688 | chr10:105192570 | lymphoblastoid | 0.004726 |
|  |  | rs6580 | chr10:105206874 | lymphoblastoid | 0.001901 |
|  |  | rs7913461 | chr10:105001325 | lymphoblastoid | 0.003924 |
| *SMC3* | chr10:112327449 | rs10261505 | chr7:154498135 | liver | 0.0002249 |
| *PAOX* | chr10:135192741 | rs4838740 | chr10:135206354 | liver | 1.67E-06 |
| *MRPL11* | chr11:66203305 | rs554202 | chr11:75428958 | liver | 0.0001535 |
| *GRTP1* | chr13:113978505 | rs2018093 | chr13:114012189 | liver | 0.003169 |
| *SERPINA11* | chr14:94908801 | rs2821096 | chr1:47742726 | liver | 0.002934 |
| *MRPL28* | chr16:417407 | rs3743888 | chr16:424420 | lymphoblastoid | 0.003523 |
| *TMEM8A* | chr16:422151 | rs3743888 | chr16:424420 | lymphoblastoid | 0.003523 |
| *PFAS* | chr17:8152637 | rs554202 | chr11:75428958 | liver | 0.0001535 |
| *AKAP10* | chr17:19809018 | rs11204303 | chr17:20244948 | lymphoblastoid | 0.00196 |
|  |  | rs119672 | chr17:19818601 | lymphoblastoid | 0.001303 |
|  |  | rs12603372 | chr17:20181566 | lymphoblastoid | 0.001555 |
|  |  | rs1638526 | chr17:19848450 | lymphoblastoid | 0.0006786 |
|  |  | rs203457 | chr17:19818338 | lymphoblastoid | 0.001719 |
|  |  | rs203466 | chr17:19809953 | lymphoblastoid | 0.001719 |
|  |  | rs203479 | chr17:19829311 | lymphoblastoid | 0.004722 |
|  |  | rs2042046 | chr17:20018841 | lymphoblastoid | 0.003067 |
|  |  | rs2108978 | chr17:19861458 | lymphoblastoid | 0.00204 |
|  |  | rs2526476 | chr17:20134289 | lymphoblastoid | 0.001555 |
|  |  | rs2526478 | chr17:20130452 | lymphoblastoid | 0.001555 |
|  |  | rs2703771 | chr17:20130154 | lymphoblastoid | 0.0009912 |
|  |  | rs2703800 | chr17:20086562 | lymphoblastoid | 0.001785 |
|  |  | rs4924803 | chr17:19991806 | lymphoblastoid | 0.003944 |
|  |  | rs4925081 | chr17:19991993 | lymphoblastoid | 0.003509 |
|  |  | rs7209653 | chr17:19882084 | lymphoblastoid | 0.0004336 |
|  |  | rs7222403 | chr17:19879164 | lymphoblastoid | 0.004331 |
|  |  | rs7223476 | chr17:20215352 | lymphoblastoid | 0.001575 |
|  |  | rs9901294 | chr17:19888501 | lymphoblastoid | 0.0007376 |
|  |  | rs9916675 | chr17:19900168 | lymphoblastoid | 0.004103 |
| *C17orf90* | chr17:79632365 | rs6565619 | chr17:79645896 | lymphoblastoid | 0.003897 |
| *ARL16* | chr17:79648507 | rs6565619 | chr17:79645896 | lymphoblastoid | 0.003897 |
| *LOC284422* | chr19:3474405 | rs1860192 | chr19:3481578 | liver | 0.00243 |
| *NDUFV3* | chr21:44324383 | rs234766 | chr21:44422273 | lymphoblastoid | 0.00076 |
|  |  | rs234773 | chr21:44425037 | lymphoblastoid | 0.0007685 |
|  |  | rs413624 | chr21:44396144 | lymphoblastoid | 0.0007661 |
|  |  | rs439861 | chr21:44404044 | lymphoblastoid | 0.0007247 |
|  |  | rs442981 | chr21:44412735 | lymphoblastoid | 0.0002253 |
| *PACSIN2* | chr22:43265777 | rs2038062 | chr22:43397966 | lymphoblastoid | 0.004262 |
|  |  | rs3788601 | chr22:43327526 | lymphoblastoid | 0.003288 |
|  |  | rs4822225 | chr22:43310652 | lymphoblastoid | 0.003386 |
|  |  | rs4822231 | chr22:43326110 | lymphoblastoid | 0.004197 |
|  |  | rs4822232 | chr22:43326807 | lymphoblastoid | 0.001378 |
|  |  | rs7364152 | chr22:43316667 | lymphoblastoid | 0.0007885 |
|  |  | rs738386 | chr22:43328419 | lymphoblastoid | 0.003062 |
|  |  | rs738387 | chr22:43329819 | lymphoblastoid | 0.000827 |
| *PACSIN2* | chr22:43265781 | rs2038062 | chr22:43397966 | lymphoblastoid | 0.004262 |
|  |  | rs3788601 | chr22:43327526 | lymphoblastoid | 0.003288 |
|  |  | rs4822225 | chr22:43310652 | lymphoblastoid | 0.003386 |
|  |  | rs4822231 | chr22:43326110 | lymphoblastoid | 0.004197 |
|  |  | rs4822232 | chr22:43326807 | lymphoblastoid | 0.001378 |
|  |  | rs7364152 | chr22:43316667 | lymphoblastoid | 0.0007885 |
|  |  | rs738386 | chr22:43328419 | lymphoblastoid | 0.003062 |
|  |  | rs738387 | chr22:43329819 | lymphoblastoid | 0.000827 |
|  | chr22:43266936 | rs3788601 | chr22:43327526 | lymphoblastoid | 0.003288 |
|  |  | rs4822225 | chr22:43310652 | lymphoblastoid | 0.003386 |
|  |  | rs4822231 | chr22:43326110 | lymphoblastoid | 0.004197 |
|  |  | rs4822232 | chr22:43326807 | lymphoblastoid | 0.001378 |
|  |  | rs7364152 | chr22:43316667 | lymphoblastoid | 0.0007885 |
|  |  | rs738386 | chr22:43328419 | lymphoblastoid | 0.003062 |
|  |  | rs738387 | chr22:43329819 | lymphoblastoid | 0.000827 |
|  | chr22:43267134 | rs2038062 | chr22:43397966 | lymphoblastoid | 0.004262 |
|  |  | rs3788601 | chr22:43327526 | lymphoblastoid | 0.003288 |
|  |  | rs4822225 | chr22:43310652 | lymphoblastoid | 0.003386 |
|  |  | rs4822231 | chr22:43326110 | lymphoblastoid | 0.004197 |
|  |  | rs4822232 | chr22:43326807 | lymphoblastoid | 0.001378 |
|  |  | rs7364152 | chr22:43316667 | lymphoblastoid | 0.0007885 |
|  |  | rs738386 | chr22:43328419 | lymphoblastoid | 0.003062 |
|  |  | rs738387 | chr22:43329819 | lymphoblastoid | 0.000827 |
|  | chr22:43272143 | rs3788601 | chr22:43327526 | lymphoblastoid | 0.003288 |
|  |  | rs4822225 | chr22:43310652 | lymphoblastoid | 0.003386 |
|  |  | rs4822231 | chr22:43326110 | lymphoblastoid | 0.004197 |
|  |  | rs4822232 | chr22:43326807 | lymphoblastoid | 0.001378 |
|  |  | rs7364152 | chr22:43316667 | lymphoblastoid | 0.0007885 |
|  |  | rs738386 | chr22:43328419 | lymphoblastoid | 0.003062 |
|  |  | rs738387 | chr22:43329819 | lymphoblastoid | 0.000827 |
|  | chr22:43272894 | rs3788601 | chr22:43327526 | lymphoblastoid | 0.003288 |
|  |  | rs7364152 | chr22:43316667 | lymphoblastoid | 0.0007885 |
|  | chr22:43275054 | rs2038062 | chr22:43397966 | lymphoblastoid | 0.004262 |
|  |  | rs4822231 | chr22:43326110 | lymphoblastoid | 0.004197 |
|  | chr22:43286953 | rs4822231 | chr22:43326110 | lymphoblastoid | 0.004197 |

**References:**

1. Asthana A, Raman B, Ramakrishna T, Rao Ch M. Structural aspects and chaperone activity of human HspB3: role of the "C-terminal extension". Cell biochemistry and biophysics 2012; 64(1): 61-72.

2. Brandl EJ, Tiwari AK, Chowdhury NI, et al. Genetic variation in the GCG and in the GLP1R genes and antipsychotic-induced weight gain. Pharmacogenomics 2014; 15(4): 423-31.

3. Siroux V, Gonzalez JR, Bouzigon E, et al. Genetic heterogeneity of asthma phenotypes identified by a clustering approach. The European respiratory journal 2014; 43(2): 439-52.

4. Pitceathly RD, Rahman S, Wedatilake Y, et al. NDUFA4 mutations underlie dysfunction of a cytochrome c oxidase subunit linked to human neurological disease. Cell reports 2013; 3(6): 1795-805.

5. Liao C, Fu F, Li R, Yang X, Xu Q, Li DZ. Prenatal diagnosis and molecular characterization of a novel locus for Dandy-Walker malformation on chromosome 7p21.3. European journal of medical genetics 2012; 55(8-9): 472-5.

6. Mori S, Kou I, Sato H, et al. Association of genetic variations of genes encoding thrombospondin, type 1, domain-containing 4 and 7A with low bone mineral density in Japanese women with osteoporosis. Journal of human genetics 2008; 53(8): 694-7.

7. de Visser MC, van Minkelen R, van Marion V, et al. Genome-wide linkage scan in affected sibling pairs identifies novel susceptibility region for venous thromboembolism: Genetics In Familial Thrombosis study. Journal of thrombosis and haemostasis : JTH 2013; 11(8): 1474-84.

8. Huang SP, Lin VC, Lee YC, et al. Genetic variants in nuclear factor-kappa B binding sites are associated with clinical outcomes in prostate cancer patients. Eur J Cancer 2013; 49(17): 3729-37.

9. Niu Z, Lei R, Shi J, et al. A polymorphism rs17336700 in the PSMD7 gene is associated with ankylosing spondylitis in Chinese subjects. Annals of the rheumatic diseases 2011; 70(4): 706-7.

10. Levesque E, Laverdiere I, Audet-Walsh E, et al. Steroidogenic germline polymorphism predictors of prostate cancer progression in the estradiol pathway. Clinical cancer research : an official journal of the American Association for Cancer Research 2014; 20(11): 2971-83.

11. Zhang LS, Yuan F, Guan X, et al. Association of genetic polymorphisms in HSD17B1, HSD17B2 and SHBG genes with hepatocellular carcinoma risk. Pathology oncology research : POR 2014; 20(3): 661-6.

12. Wu CH, Yang JG, Chang YJ, Hsu CC, Kuo PL. Screening of a panel of steroid-related genes showed polymorphisms of aromatase genes confer susceptibility to advanced stage endometriosis in the Taiwanese Han population. Taiwanese journal of obstetrics & gynecology 2013; 52(4): 485-92.

13. Bergholdt R, Brorsson C, Palleja A, et al. Identification of novel type 1 diabetes candidate genes by integrating genome-wide association data, protein-protein interactions, and human pancreatic islet gene expression. Diabetes 2012; 61(4): 954-62.

14. Zhao YY, Zhang FJ, Zhu SQ, et al. The association of a single nucleotide polymorphism in the promoter region of the LAMA1 gene with susceptibility to Chinese high myopia. Molecular vision 2011; 17: 1003-10.

15. Perry JR, Voight BF, Yengo L, et al. Stratifying type 2 diabetes cases by BMI identifies genetic risk variants in LAMA1 and enrichment for risk variants in lean compared to obese cases. PLoS genetics 2012; 8(5): e1002741.

16. Varade J, Comabella M, Ortiz MA, et al. Replication study of 10 genes showing evidence for association with multiple sclerosis: validation of TMEM39A, IL12B and CBLB [correction of CLBL] genes. Multiple sclerosis 2012; 18(7): 959-65.

17. Kariuki SN, Franek BS, Kumar AA, et al. Trait-stratified genome-wide association study identifies novel and diverse genetic associations with serologic and cytokine phenotypes in systemic lupus erythematosus. Arthritis research & therapy 2010; 12(4): R151.

18. Campbell CD, Mohajeri K, Malig M, et al. Whole-genome sequencing of individuals from a founder population identifies candidate genes for asthma. PloS one 2014; 9(8): e104396.

19. Svensson-Farbom P, Wahlstrand B, Almgren P, et al. A functional variant of the NEDD4L gene is associated with beneficial treatment response with beta-blockers and diuretics in hypertensive patients. Journal of hypertension 2011; 29(2): 388-95.

20. Nakamura M, Miyachi Y. Congenital hyponychia without RSPO4 mutation. Acta dermato-venereologica 2008; 88(5): 511-2.

21. Kohsaka T, Yuan ZR, Guo SX, et al. The significance of human jagged 1 mutations detected in severe cases of extrahepatic biliary atresia. Hepatology 2002; 36(4 Pt 1): 904-12.
